# Regulator of G protein signaling 10: a critical regulator of chronic systemic inflammation in parkinson’s disease

**DOI:** 10.1186/s12974-026-03858-4

**Published:** 2026-05-22

**Authors:** Janna E. Jernigan Posey, Kelly B. Menees, Malú Gámez Tansey

**Affiliations:** 1 Center for Translational Research in Neurodegenerative Disease, College of Medicine, University of Florida, Gainesville, FL USA; 2 Department of Neuroscience, College of Medicine, University of Florida, Gainesville, FL USA; 3 McKnight Brain Institute, University of Florida, Gainesville, FL USA; 4 Aligning Science Across Parkinson’s (ASAP) Collaborative Research Network, Chevy Chase, MD USA; 5 Department of Neurology, Indiana University School of Medicine, Indianapolis, IN USA; 6 Stark Neuroscience Research Institute, Indianapolis, IN USA

**Keywords:** Chronic Systemic Inflammation, Immune Dysregulation, Neuroinflammation, Regulator of G Protein Signaling 10 (RGS10), Neurodegenerative Diseases, Parkinson’s Disease

## Abstract

The inflammatory response mounted by the immune system is a protective process necessary for survival following exposure to insults. However, it must be tightly regulated to maximize efficient elimination of pathogens and to minimize collateral damage to the host. Failure to maintain balance and resolve an inflammatory response can result in chronic systemic inflammation (CSI). CSI has been identified as a key factor in the pathogenesis of many diseases including neurodegenerative diseases (NDs). This review covers the growing relationship between CSI and NDs with a particular emphasis on Parkinson’s disease (PD). Furthermore, we discuss the role of an important negative regulator of inflammatory responses: Regulator of G-protein Signaling-10 (RGS10), within the context of CSI and PD.

**Graphical Abstract:**

**Figure:**
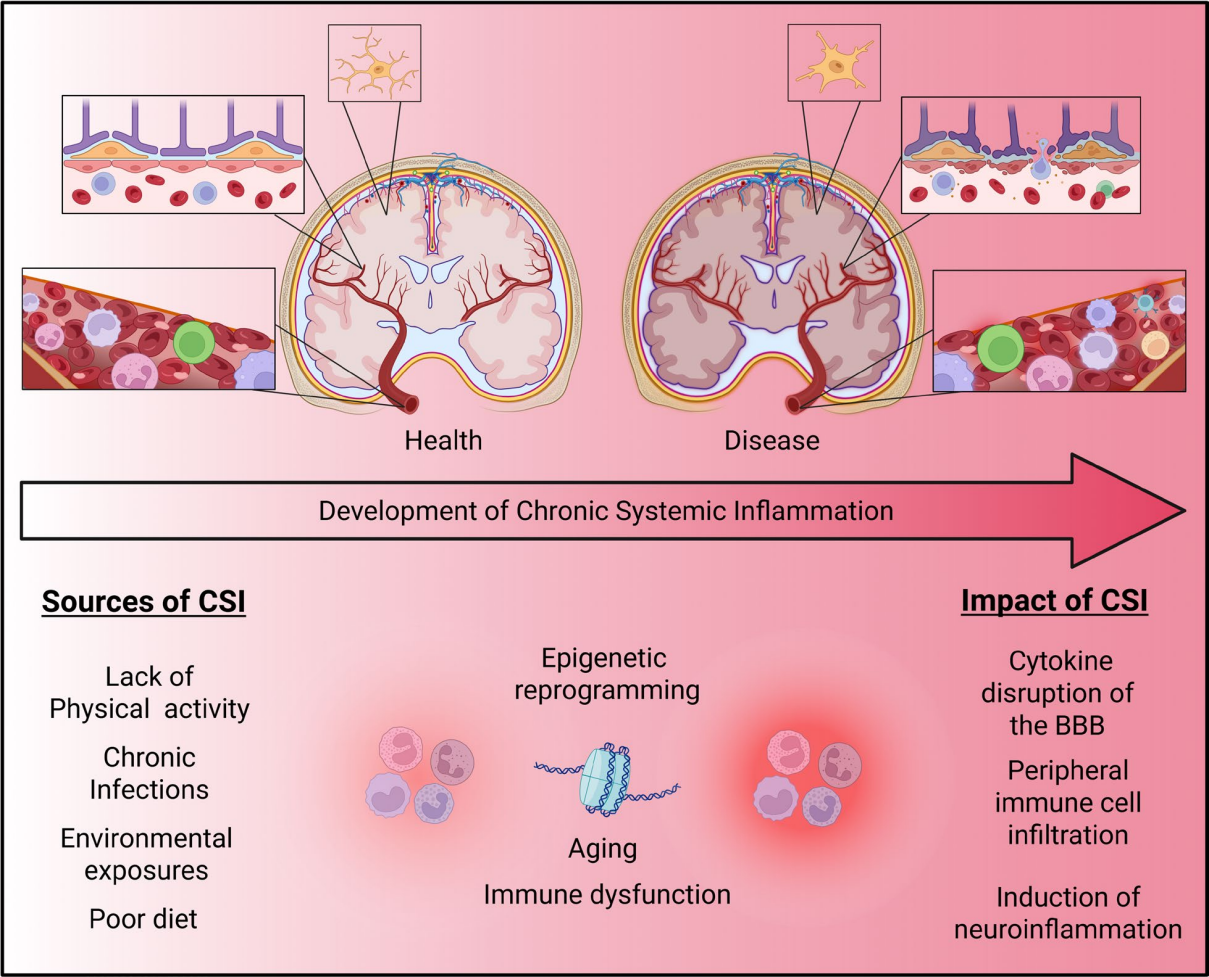
CSI contributes to disrupted CNS homeostasis and the development of neurodegenerative diseases. Environmental exposures and lifestyle choices combine to induce CSI, while CSI and aging reciprocally influence the downregulation of protective factors like RGS10, thereby promoting peripheral immune dysregulation that contributes to neuroinflammation and disrupted CNS homeostasis and disease. Created with BioRender.com

## Introduction

To defend the body from injurious agents, such as pathogens, the body employs the process of acute inflammation. Once activated by the presence of an immunogen, the innate immune system will orchestrate a collective strike with the adaptive immune system to eliminate the threat and remodel injured tissue [1]. The ability for the immune system to clear threats is crucial to host survival. However, inflammatory responses are a heavy drain on host resources and utilize weaponry capable of inflicting collateral damage to the host [2, 3]. Therefore, the inflammatory response must be tightly regulated. Failure to resolve the inflammatory response results in chronic inflammation, which can be deleterious to tissue and maintenance of physiological processes [4].

Chronic inflammation that is found throughout the body, known as chronic systemic inflammation (CSI), has been implicated in multiple diseases [5]. Interestingly, studies have identified an association of CSI with neurogenerative diseases (NDs) such as Alzheimer’s Disease (AD) and Parkinson’s disease (PD), illustrating the potential for CSI to impact the development/progression of diseases not only in the periphery but also in the brain [5, 6]. Notably, known sources of CSI have been identified as risk factors for developing NDs and clinical data have revealed an abundance of peripheral immune cell alterations in NDs, including PD, that evince the presence of CSI [5, 7–14]. Moreover, CSI may be capable of reprogramming immune cells to downregulate protective factors thereby enhancing the cycle that sustains and propagates CSI [15].

Of particular interest, is the protective factor known as regulator of G-protein signaling-10 (RGS10). RGS10 is a homeostatic regulator of immune cells, acting as a negative regulator of pro-inflammatory responses primarily in myeloid cells [16–19]. Yet, RGS10 has been shown to be downregulated upon inflammatory stimuli which may be responsible for the decreased levels of RGS10 have been reported in immune cells of individuals with PD [16–22]. Moreover, this decrease in RGS10 may relate to functional consequences within the development of PD as preclinical models have identified hallmarks of parkinsonian pathology such as nigral degeneration and microgliosis in RGS10-deficient animals exposed to CSI, while the lentiviral-mediated introduction of RGS10 into a parkinsonian rat model prevented dopaminergic cell death and neuroinflammation in the substantia nigra [17, 20].

In this review we cover the development of CSI and its contributions to NDs, with a particular emphasis on PD. Moreover, we review the role of RGS10 as a protective factor, that is likely lost because of chronic, low-grade, sterile inflammation that develops with age known as inflammaging [23], but also as a promising therapeutic target that could mitigate the effects of CSI to reduce risk of NDs.

### Chronic systemic inflammation

To ensure survival and re-establish homeostasis upon infection or injury, the body utilizes inflammation, a protective process designed to eliminate insults and facilitate tissue repair. When dysregulated or unbalanced, however, the inflammatory response shifts from a protective to a disease promoting system. For example, minimal or insufficient immune responses leave infections unrestricted and capable of actively killing host cells, monopolizing critical resources, and multiplying until the host organism is overwhelmed. As such, individuals who are immunocompromised may experience common infections such as the flu as potentially life-threatening events [24]. On the other hand, excessive or prolonged inflammatory responses are deleterious to host tissues as well and can help promote future chronic diseases [5].

As the immune system can promote disease when hyper- or hyporeactive, the extent and timing of an inflammatory response must be tightly regulated in a multitude of ways. Firstly, the immune response must be regulated in both its amplitude and localization. Failure to contain an inflammatory response to the injury site (i.e. local inflammation) can result in a systemic response (systemic inflammation) and if this inflammatory response is unchecked in amplitude, patients can become septic, a life-threating acute inflammatory response [25]. Secondly, inflammation must be regulated temporally. As mechanisms for the inflammatory response to clear infections are also capable of harming host cells it is imperative to maintain an acute response that maximizes elimination of injurious agents while minimizing collateral host cell damage [26]. Persistent and unresolved inflammation is known as chronic inflammation which damages tissues and the overall health of the organism[5].

**Fig. 1:**
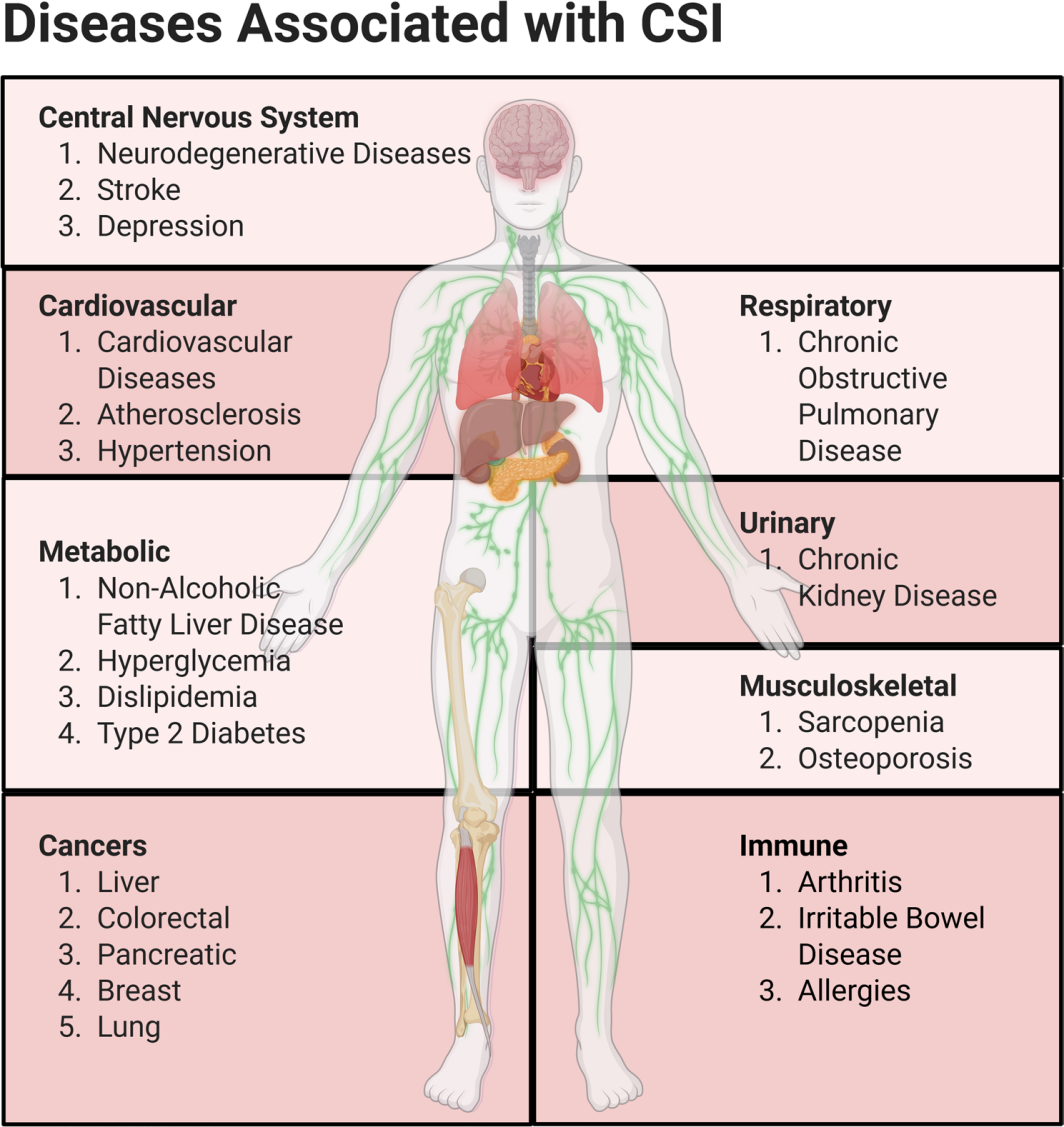
Diseases associated with CSI. CSI drives the development and progression of significant diseases throughout the body, including neurodegenerative diseases. Created with BioRender.com

Chronic systemic inflammation (CSI) is defined as a persistent low-grade inflammatory response present throughout the body that ultimately leads to collateral tissue damage [5, 27]. CSI has gone greatly underappreciated in the understanding of disease, the first mention of the term in the literature occurring in the year 2000 [28], and only recently gaining more attention in the field of neuroscience. However, in the last 20 years, CSI has been identified as a central driver for the development and progression of a multitude of diseases [5, 29, 30] (Fig. 1). CSI mediates the leading causes of death and disability worldwide and is recognized as the greatest threat to human health by the World Health Organization [4].

CSI can be recognized through the development of multiple symptoms that may include body pain, arthralgia, myalgia, chronic fatigue and insomnia, depression, anxiety and mood disorders, gastrointestinal complications like constipation, diarrhea, and acid reflux, weight gain or weight loss, and or frequent infections [4]. Moreover, CSI results in exacerbation of cognitive decline after inflammatory insults such as urinary tract infection and surgery in ND patients [31–33].

On a molecular level, CSI is generally triggered by DAMPs in the absence of an active infection or injury while reactive oxygen species (ROS) produced in the inflammatory response act as a central generator of collateral damage [5, 26, 34]. Levels of chemokine ligand 9 (CXCL9), C reactive protein (CRP), and tumor necrosis factor (TNF) in the blood have recently been proposed as reliable markers of active CSI in aging populations [35, 36]. Correspondingly, meta-analyses have identified elevated levels of CRP and TNF in the blood and CSF in PD and elevated levels of CXCL9 and TNF in the blood of individuals with AD [37, 38]. However, to date, a single biomarker of CSI has yet to be determined. The discernment of the ‘immunome’, which would characterize the whole of cellular and molecular immunologic changes for patient populations, may offer a more cohesive delineation of CSI [35]. On a cellular level, we can observe specific changes in the white blood cell composition, such as the replacement of neutrophils by macrophages, lymphocytes, and plasma cells that mass produce inflammatory mediators, signifying CSI [4]. In NDs, such as PD, clinical data have revealed a plethora of peripheral immune alterations such as an increase of pro-inflammatory cytokines and pro-inflammatory innate and adaptive immune cell subsets in the circulation and cerebral spinal fluid (CSF) and a rise in central nervous system (CNS) infiltrating peripheral immune cells [7–12]. Specifically, B and T cell populations in PD shift away from naïve populations towards more effector/memory populations while neutrophils to lymphocyte ratios increase [39]. Peripheral monocyte dynamics also demonstrate elevated inflammatory subsets and responses; however, a few studies have shown an overall lack of response of PD monocytes as well [40–43].

The molecular and cellular changes in CSI can result from unresolved acute inflammatory responses, chronic exposure to low-level irritants, or immune dysregulation [4]. These contributing agents are a common consequence of environmental exposures, lifestyle choices, genetics, as well as the aging process [5, 15]. Specifically, diet, the gut microbiome, physical inactivity, stress, disturbed sleep, exposure to industrial toxicants, genetics, aging, and pre-existing chronic inflammatory diseases (CIDs) such as rheumatoid arthritis (RA), inflammatory bowel disease (IBD), irritable bowel syndrome (IBS), diabetes, non-alcoholic fatty liver disease (NAFLD), and psoriasis have been identified as common sources for CSI [5]. It is important to note that sources of chronic inflammation overlap significantly with known risk factors for developing NDs such as PD. In fact, due to the idiopathic nature of NDs like AD and PD, the accumulation of compounding genetic and environmental risk factors has gained acceptance as a hypothesis for the development and progression of NDs [9]. Here, we highlight chronic inflammation as a shared downstream consequence of such genetic and environmental risk factors, signifying the capacity of CSI as a key target for therapeutic intervention in NDs.

Diving deeper into the sources of CSI, we observe that levels of lipopolysaccharide (LPS) are often elevated in the blood [44]. Lipopolysaccharide (LPS), also referred to as endotoxin, is a potent immunogen capable of inducing CSI once in circulation [45]. LPS is a significant component of the outer-cell membrane of gram-negative bacteria, which is shed by living bacterium or released by dead bacterium and therefore can trigger metabolic endotoxemia [46]. Elevated LPS levels have been demonstrated in the blood of individuals from multiple CIDs such as IBD, NAFLD, and diabetes [47–49]. LPS is a powerful activator of immune cells that signal through toll like receptors [50, 51]. LPS-producing bacteria live primarily in the gut [52] Therefore, conditions that disturb the gut microbiome and promote a leaky gut such as consuming a western diet, sedentary lifestyle, excessive/chronic stress or alcohol consumption, and certain medications have been found to increase LPS levels in the blood [44, 53–59]. Analyses of the gut microbiome of PD patients have revealed an increase in the number of LPS-producing species compared to healthy controls [51, 60, 61]. Moreover, multiple studies have reported increased levels of LPS in the blood of PD, AD, and ALS patients [51, 62]. Elevated levels of endotoxin have also been reported in the CSF of individuals with AD compared to healthy controls [62]. In humans, intravenous (IV) injection of 1ng/kg of LPS induced elevated levels of inflammatory cytokines in the blood, elevated heart rate, fever, sickness symptoms, and robust microglial activation within 3 h of injection [63]. Additionally, a single IV injection of 0.2ng/kg of LPS resulted in a negative association between circulating IL-6 and memory functions independent of physical stress symptoms [64]. These studies demonstrate that small amounts of LPS are sufficient to induce systemic inflammation that can impact the CNS in humans.

Pre-clinical models are necessary to further our understanding of CSI in the development and progression of ND pathology. CSI is commonly modeled with LPS injections [65]. In mice, high doses of LPS (5 mg/kg) injected into the peritoneum have been shown to induce dopaminergic degeneration with aging [66–69]. Mouse models that include secondary hits, such as PD relevant genetic mutations, see evidence of neuronal loss at earlier timepoints [70]. However, a significant aspect of CSI that these models fail to encapsulate is its chronic low-grade nature. Most reported LPS paradigms are limited to either a single dose or up to one week of daily injections [46]. Interestingly, there are multiple depression studies that utilize LPS paradigms that last for multiple weeks [71, 72], while only three studies to our knowledge that have assessed neurodegenerative outcomes of the nigrostriatal pathway utilize these longer LPS paradigms [20, 73, 74]. Importantly, these studies have shown that the induction of low-grade inflammation over the course of months coupled with secondary hits are sufficient to induce dopaminergic degeneration and neuroinflammation [20, 73, 74]. Another study utilizing 0.5 mg/kg intraperitoneal (IP) LPS once per week for 4 weeks did not assess neuronal survival but did see increased infiltration of neutrophils in the brain up to 8 h following the last injection, suggesting that peripheral immune infiltration may play an active role in the development of neuroinflammation and neurodegeneration as a result of CSI [75]. Moreover, Frank-Cannon et al. reported that prolonged low-grade LPS exposure (6 months) starting in young adulthood was sufficient to promote dopaminergic dysfunction in WT mice without a secondary hit [73]. The combination of human and preclinical data has resulted in the rise of the endotoxin hypothesis for PD and AD, suggesting that endotoxin induced CSI may be a primary driver for the development of NDs [51, 62].

Current research into how CSI impacts the CNS in pre-clinical models, however, is still limited, as most studies focus on how acute, almost septic, inflammatory responses affect the brain [76]. Moreover, the most common model of systemic inflammation, IP LPS injections, lack standardization due to the absence of reporting dosages in endotoxin units (which differ by lot) instead of grams per kilogram, which significantly hinders the ability of the field to not only interpret but also to replicate study results. Lastly, studies that incorporate aged animals are few and far between, despite the fact that age is the number one risk factor for age-related neurodegenerative conditions like PD.

### Development of immune dysregulation in CSI

**Fig. 2:**
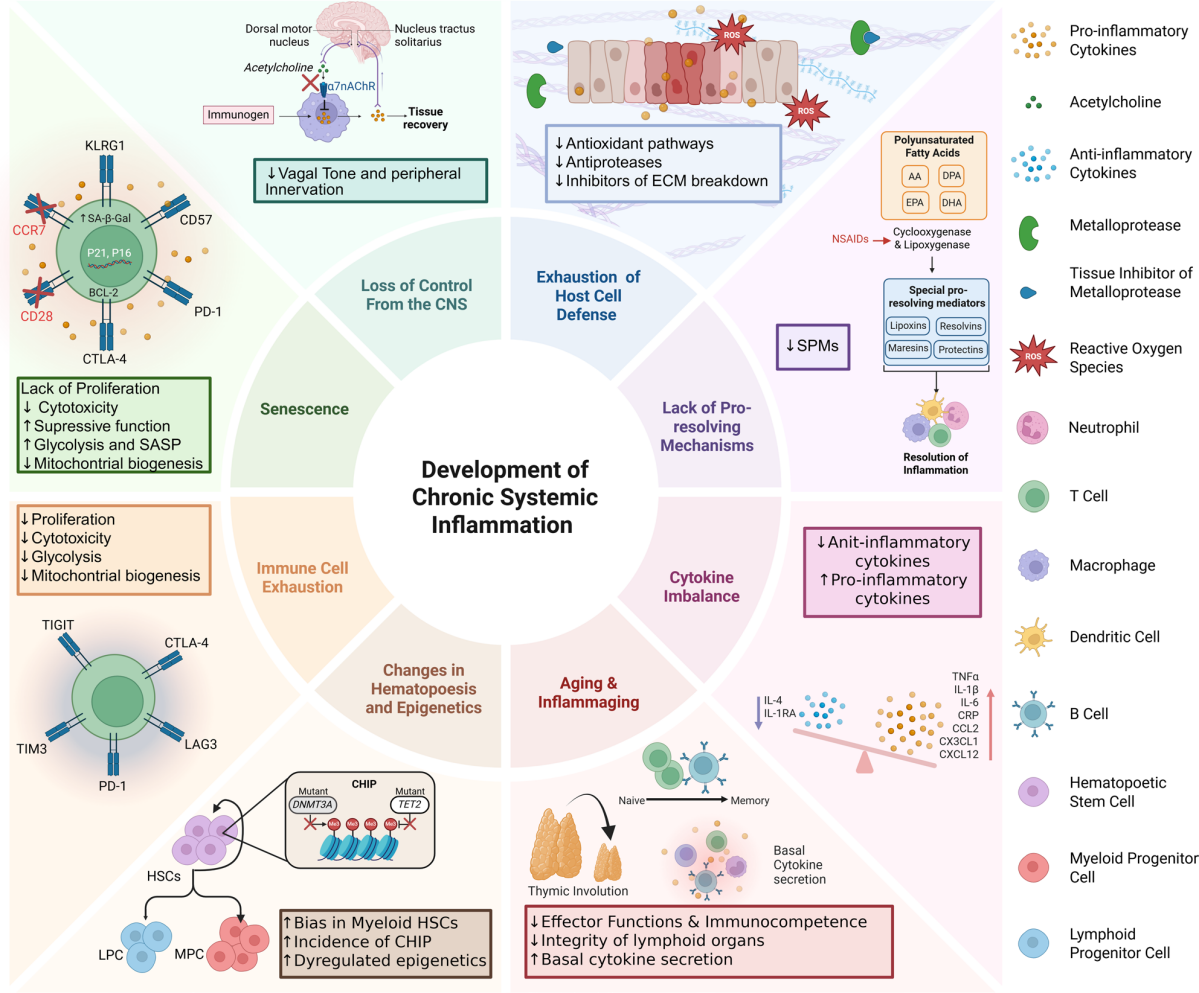
Immune dysregulation leads to the development of chronic systemic inflammation. Chronic inflammatory responses occur due to the inability to internally resolve the inflammatory response, continued presence of DAMPs due to overwhelmed host protective mechanisms and cell death, imbalanced pro- and anti-inflammatory signaling protein expression, loss of regulation from the brain-body circuit, as well as emergent phenomena that come with aging, including the development of senescent cells, exhausted immune cells, inflammaging, altered hematopoietic stem cells (HSC) differentiation, epigenetic modifications, and decreased integrity of lymphoid organs. Created with BioRender.com

As mentioned above, CSI can occur due to chronic exposure to low-level irritants, or immune dysregulation. Here, we will detail how specific types of immune dysregulation contribute to the development of CSI (Fig. 2).

#### Lack of resolution

As previously established, CSI occurs under sterile conditions in which the injury or infection has already been cleared and yet the inflammatory response has not resolved, highlighting the importance of active pro-resolving anti-inflammatory mechanisms built into the immune system. A fundamental pro-resolving mechanism found within the immune system, involves anti-inflammatory cytokines. Anti-inflammatory cytokines are released to prevent overactivation of immune cells during an immune response and promote wound healing once the threat has been cleared [77]. In PD, cytokine production becomes unbalanced with an overall reduction in circulating anti-inflammatory cytokines, and a reciprocal increase in circulating pro-inflammatory cytokines [8]. Specifically, studies have found decreased levels of IL-4 and IL-1RA in the blood of individuals with PD compared to age-matched healthy controls [8]. However, studies have also identified an increase in circulating IL-10 in PD, which is likely compensating for the stark increase of pro-inflammatory mediators [8]. This imbalance of anti-inflammatory and pro-inflammatory cytokines contributes to the feed-forward cycle of CSI.

Over the last two decades, research dedicated to understanding the resolution phase of inflammation has uncovered a class of polyunsaturated fatty acid (PUFA) derivatives termed specialized pro-resolving mediators (SPMs) [78]. SPMs prevent neutrophil infiltration and induce macrophage efferocytosis (clearance of apoptotic cells) to pump the brakes on the inflammatory response and enhance wound healing [79]. The catalysis of PUFAs (EPA, DPA, and DHA) into SPMs (resolvins, protectins, lipoxins, and maresins) is dictated by two oxygenases, cyclooxygenase-2 (COX-2) and lipoxygenase (LOX) [80]. While COX-2 is a potent generator of pro-inflammatory prostaglandins in the acute phase of inflammation, specific drugs such as aspirin and non-steroidal anti-inflammatory drugs (NSAIDs) add post-translational modifications to COX-2 and shift its enzymatic activity to the production of SPMs [81]. COX-2 will shift to anti-inflammatory prostaglandin and SPM production after the acute phase of the inflammatory response via a currently unknown mechanism [81]. Moreover, LOX can be induced by IL-4 and IL-13 cytokines secreted from the TH2 helper T cell subset [82]. SPMs and their receptors have been found to be decreased in most CIDs including NDs [80]. Several kinds of pro-resolving mimetics are currently under clinical trials for CIDs and have shown pre-clinical efficacy at nanogram concentrations [80]. Furthermore, the protectin PD1 has been shown to blunt neuroinflammation and prevent early pathology in a rat model of PD [83]. In summary, disrupting pro-resolving mechanisms of the inflammatory response leaves the door open for promotion of chronic inflammation and the development of CIDs.

In tandem with the need for internal pro-resolving mechanisms within immune cells, host cells invoke protective mechanisms to bolster themselves and increase their threshold for inflammation driven toxicity. Host cells are capable of antagonizing multiple cytotoxic mediators released from immune cells, which are reviewed elsewhere [27]. If these protective mechanisms of the host cells are overwhelmed due to a severe or long-lasting immune responses, host cells will succumb to the toxic environment and promote the persistent nature of chronic inflammation.

#### Aging

Major changes to the immune system occur with age. On a cellular level, in aging, cells throughout the body, including immune cells, are subject to genomic instability, telomere attrition, loss of proteostasis, epigenetic alterations, impaired macroautophagy, dysregulated nutrient sensing, mitochondrial dysfunction, senescence, stem cell exhaustion, and altered intercellular communication [84]. These phenomena have been designated as the hallmarks of aging, all of which can contribute to the development of or can be induced themselves by chronic inflammation, highlighting the inflammatory nature of the aging process, a phenomenon described as inflammaging [15, 85]. Inflammaging is a shift of both innate and adaptive immune cells to a chronically activated inflammatory state [86]. The existence of inflammaging can be demonstrated by the increased incidence of proinflammatory mediators such as TNF, IL-6, CRP, IL-8, and CXCL9 in aged individuals [23]. Additionally, the development of clonal hematopoiesis of indeterminate potential (CHIP), a bias for myeloid differentiation in hematopoietic stem cells (HSCs), and accumulation of senescent cells is observed [87–89]. All three of these phenomena are directly linked to changes in the regulation of the epigenetic landscape of immune cells and contribute to chronic inflammation [88, 90, 91]. CHIP is a condition defined as the acquisition of leukemogenic mutations in HSCs. The incidence of CHIP increases with age and exposure to chronic inflammation and has also been associated with the development of cardiovascular disease [92]. The most common mutations that occur in CHIP are found in DNMT3A and TET2, both of which participate in DNA methylation [88]. Mutated forms of DNMT3A and TET2 help promote self-renewal of HSCs, through hypomethylation DNA regions associated with HSC activity and hypermethylation of DNA regions related to progeny cells respectively [88]. Further alterations in HSC can be appreciated by the general reduction of hematopoietic output and bias toward myeloid lineage cell types [91, 93]. Interestingly, targeting myeloid biased HSCs and senescent cells has shown promise in restoring more youthful immune cell dynamics and expanding healthy aging in preclinical models respectively [94–96]. We will cover senescence in more detail in the following sections.

On the tissue level, the process of thymic involution is particularly important to consider in immune cell aging. Thymic involution is the process in which the thymus, a primary lymphoid organ responsible for producing immunocompetent lymphocytes, atrophies with age [97]. Thymic involution is an evolutionary process that conserves host resources once the T cell repertoire has been established in an adult organism [97]. As such, a shift from antigen naive to memory cell lymphocyte populations occurs with age, with decreases in CD8+ naïve T cells identified as a hallmark of immune aging in humans [86, 98]. Consequences of this phenomenon, include decreased immunocompetence and diversity of T and B cell repertoires, resulting in defective lymphocyte function ultimately leading to decreased ability to fight novel infections, increased incidence of autoimmunity, and decreased tumor surveillance [97, 99]. Furthermore, immune cell exhaustion, primarily studied in T cells, has been found to accumulate with aging and exposure to a tumor microenvironment or persistent antigen stimulation, such as in CSI [100, 101]. Due to increased inhibitory signaling through major receptors such as programmed cell death protein 1 (PD-1) and T-cell immunoglobulin and mucin-domain containing-3 (TIM-3), primary markers of exhausted T cells, T cells largely become hyporesponsive, have reduced cytotoxicity, proliferation, glycolysis, and mitochondrial biogenesis [102], ultimately magnifying age related decreases in immunocompetence. The innate immune system has also been observed to change with age. Specifically, alterations in the ability of major innate immune cell types to participate in chemotaxis, phagocytosis, antigen presentation, and killing occur with age signifying an overall reduction of immune functionality in aging [23]. In summary, aging can act as source of intrinsic immune dysregulation characterized by decreased functionality of the immune system and the development of inflammaging.

#### Senescence

Senescence has been identified as one of the 12 hallmarks of aging and has been implicated in the development of age-related diseases such as NDs [15, 84]. Cells that have lost their ability to proliferate indefinitely due to accumulation of cellular damage are considered senescent [103]. Senescence occurs in a multitude of processes from development, to wound healing, to aging and primarily occurs because of DNA damage [103]. While senescence can have biologically beneficial outcomes in development and wound healing, prolonged senescence, such as in aging, can result in chronic inflammation due to a key characteristic of senescent cells called senescence associated secretory phenotype (SASP) which involves the release of cytokines, chemokines, growth factors, and proteases [103, 104]. This is particularly evident in immune cells that become senescent in a process termed immuno-senescence [104]. Within NDs, increased numbers of senescent cells within the brain have been reported [105].Moreover, senescence exists in a bidirectional relationship with CSI, as CSI can induce cellular senescence perpetuating a positive feedback loop of deleterious CSI [15, 106].

#### Epigenetics

Moreover, as with cellular senescence, hematopoietic stem cell bias, and clonal expansion, we see that CSI can alter immune cells and immune dysregulation can be introduced via the process of epigenetic reprogramming [15, 107, 108]. Epigenetics can be defined as changes in gene expression that occur without altering the DNA sequence. The most well studied epigenetic regulators are post-translational modification to the chromosomal support proteins, histones, and DNA itself, specifically through acetylation and methylation. DNA methylation patterns have been shown to be a reliable predictor of the chronological age of an organism and serve as the basis for the development of epigenetic clocks [109]. In aging, hypomethylation of DNA, which permits enhanced gene expression, has been widely reported in conjunction with increased hypermethylation on certain CpG island promoters that cause gene silencing [110]. Accordingly, enhanced chromatin accessibility has been reported in gene regions associated with inflammation in immune cells of aging humans [111]. Moreover, DNA methylation changes have been identified in CIDs, acting on multiple inflammatory pathways [112]. Changes in histone acetylation also significantly contribute to longevity and inflammation. In aging, activity and expression levels of certain sirtuins, a class of histone deacetylases (HDACs) that enhance genomic stability and help inhibit senescence, are decreased [113] while histone acetyltransferase (HAT) Sas2, which silences telomeric repeats, is increased [114] consequently promoting senescence and telomere attrition. Histone acetylation has also been shown to regulate nuclear factor kappa-light-chain-enhancer of activated B cell (NFκB) dependent inflammation [115] and changes in histone acetylation and methylation have been associated with multiple CIDs such as heart disease, AD, type 2 diabetes, psoriasis, asthma, systemic lupus erythematosus, and multiple sclerosis [112]. So, while epigenetic modifications are abundant in aging and CIDs, the pro-inflammatory environment generated by such epigenetic modifications can induce further epigenetic remodeling.

The impact of inflammation on the epigenetic landscape can be appreciated from multiple different perspectives. For example, a large epigenome-wide association study of more than 22,000 individuals indicated that altered CpG methylation was a consequence of elevated levels of CRP in the blood [116]. Moreover, studies addressing the concept of immune tolerance have demonstrated LPS-induced epigenetic alterations in acute and non-persistent inflammatory conditions [117]. Foster et al. identified that LPS stimulation of bone marrow derived macrophages reduced histone acetylation at pro-inflammatory genes but not antimicrobial genes in tolerized macrophages, highlighting that epigenetics is a critical factor in immune responses to repeated insults [117]. Persistent low-dose LPS administration, however, has been shown to induce epigenetic changes that result in low-grade inflammatory murine monocytes [107]. Interestingly, persistent high does LPS administration instead induced exhaustive immune phenotypes [107]. This suggests that CSI may downregulate protective factors within the immune system that ultimately lead to immune dysregulation and the persistence of CSI which may contribute to the pathogenesis of NDs.

#### Loss of control from CNS

In the early 2000’s trailblazing studies uncovered the cholinergic anti-inflammatory reflex, a reflex in which the vagus nerve detects and responds to peripheral cytokines with the release of acetylcholine that acts on α7nAChR receptor to inhibit further cytokine release [118, 119]. We have recently gained new mechanistic insights into this body-brain circuit that helps regulate the immune response [120]. Specifically, nuclei within the vagal nerve both respond to and influence the inflammatory response [120]. Ablating the body-brain circuit during an inflammatory challenge increased the inflammatory response, while stimulating vagal nuclei during an inflammatory challenge, dampened the pro-inflammatory response [120]. Disrupted signaling in this pathway may result in top-down immune dysregulation. PD is accompanied by vagal nerve atrophy and autonomic dysregulation that impact the cardiovascular, gastrointestinal, genitourinary, and thermoregulatory systems [121, 122]. Moreover, peripheral cholinergic denervation assessed through positron emission tomography (PET) radiotracer ^11^C-donepezil, which bind directly to acetylcholinesterase, was observed in the intestine of individuals with PD [123], suggesting that the cholinergic anti-inflammatory reflex may be disrupted in PD.

### Neuro-immune crosstalk in CSI

As substantiated above, a dysregulated immune system will not only succumb to CSI inducing events but may also amplify its effects. In the CNS, CSI may generate several different routes of influence including paracrine and endocrine signaling of cytokines or infiltrating peripheral immune cells [124]. It has been demonstrated that cytokines such as IL-1β, IL-6, and TNF have saturable blood-brain transport across the blood-brain barrier (BBB) [125]. Cytokines can also induce BBB disruption by reducing tight junction proteins, effectively allowing for more cytokine influx into the CNS [124]. BBB leakage has been demonstrated in individuals with PD utilizing dynamic contrast enhanced magnetic resonance imaging (DCE-MRI) which allows for assessment of more subtle or chronic BBB disruption [126]. These same imaging techniques have elucidated significant and early BBB leakage in individuals with AD as well [127, 128].

**Fig. 3:**
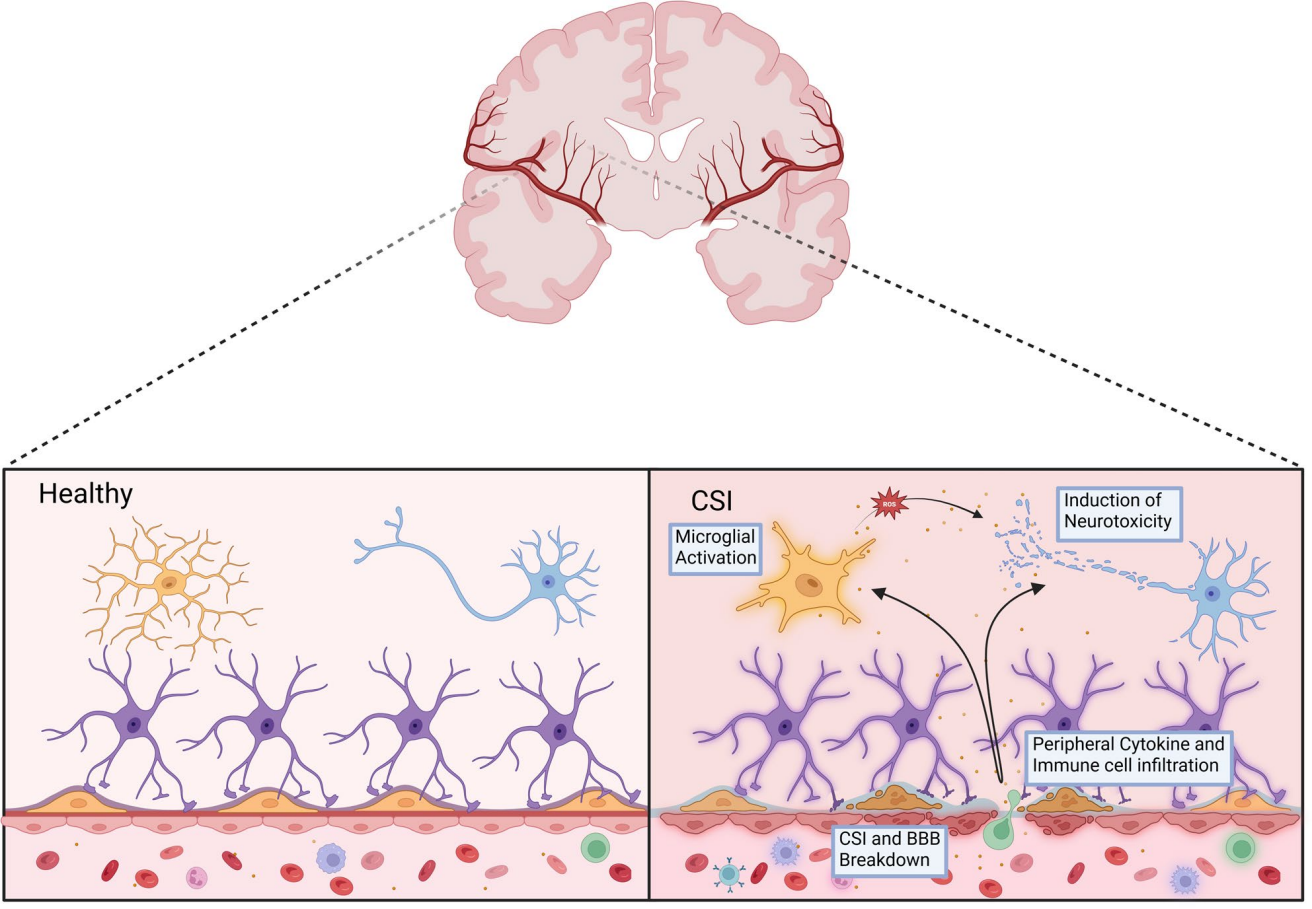
Neuroimmune Crosstalk in CSI: Elevated circulating cytokine levels can disrupt the BBB. Leaky BBB allows for the infiltration of peripheral cytokine and immune cells into the brain and the induction of neuroinflammation. Created with Biorender.com

Once in the CNS, cytokines can activate the innate resident immune cells, microglia, and initiate neuroinflammation [129]. Post-mortem imaging and omics studies as well as anti-mortem PET imaging studies have revealed chronically activated microglia within NDs that contribute to neuroinflammation [130–135]. Moreover, studies have shown peripheral infiltration of immune cells in the brains of PD patient tissue [136]. However, it is unknown whether patients with CIDs experience increased peripheral immune cell infiltration into the CNS. It has been recently discovered that immunologic niches exist within the CNS, including the choroid plexus, the meningeal lymphatic system, and the cerebral spinal fluid (CSF), where peripheral immune cells actively participate in immune surveillance [137–139]. Additionally, bone marrow-derived immune cells are essential in neuroprotection, plasticity, and in repair outside of disease, in the context of acute sterile CNS injury [138]. Nevertheless, dysfunctional responses of bone marrow derived immune cells can still contribute to tissue damage and disease progression once in the CNS as neurons are particularly sensitive to inflammatory products such as ROS [140] (Fig. 3). The concept of neuronal vulnerability to systemic inflammation can especially be appreciated by the fact that large retrospective studies have found a significant reduction in the number of individuals with CIDs such as RA and IBD who develop neurodegenerative conditions like PD and AD when taking anti-TNF biologics when compared to those who did not [141, 142].

### Current and future strategies for combating CSI in PD

Unsurprisingly, curtailing deleterious inflammatory processes in neurodegenerative diseases has gained interest as a therapeutic strategy. The AD field has progressed to investigate neuroimmune/inflammatory targets in clinical trials faster than the PD field with approximately 17% of clinical trials (24 studies) dedicated to inflammatory targets as of 2025 [143]. While only approximately 5% of clinical trials (8 studies) were targeting inflammation in Parkinson’s disease as of 2024 [144–146]. Primary immune targets in clinical trials for PD include Sargramostim, a human granulocyte macrophage colony stimulating factor (GMCSF) that can induce T regulatory responses, Montelukast, a repurposed asthma drug, NE3107, a brain penetrant extracellular signal-regulated kinase (ERK) inhibitor in macrophages, AKST4290, a C-C chemokine receptor type 3 (CCR3) antagonist, Exenatide and Lixisenatide, which are GLP-1 agonists, and multiple NLR family pyrin domain containing 3 (NLRP3) inhibitors [145–147]. Most of these targets are still in early phases of clinical trials except for Montelukast and GLP-1 agonists. A phase 2/3 study for Montelukast was completed as of December 2025 and still awaits results from the study. Exenatide, completed phase 3 clinical trials in the United Kingdom and did not see any disease modifying effects via the MDS-Unified Parkinson’s disease rating scale (UPDRS) III movement scores measurement [147]. Lixisenatide, however, has completed phase 2 trials and found that 12 months of treatment with Lixisenatide in individuals with early PD resulted in decreased progression of motor disability compared to placebo [146].

Further investigation into endogenous negative regulators of inflammation may also prove beneficial for future therapeutic strategies. A few such negative regulators that have been investigated in PD include tumor necrosis factor α-induced protein 3 (TNFAIP3), CD200-CD200R1, and RGS10 [148–150]. TNFAIP3 also known as A20 is a deubiquitinase that inhibits activation of proteins upstream of the IKK complex that is necessary for disinhibition and nuclear translocation of NFκB [151]. In PD, reduced levels of A20 have been reported in the blood and substantia nigra [152, 153]. Levels of A20 were also reduced in PARK2 PD patient macrophages and negatively correlated to NLRP3 mediated Interleukin 1 beta (IL-1β) release, indicating that Park2 mutations may enhance NLRP3 activation via attenuating A20 expression [154]. Moreover, A20 was found to be neuroprotective in the 1-Methyl-4-phenyl-1,2,3,6-tetrahydropyridine (MPTP) neuro-toxicant mouse model of PD by restricting NFκB and the mechanistic target of rapamycin (mTOR) pathways [153].

The CD200-CD200R1 axis is considered an inhibitory signaling pathway for myeloid cells. This can be highlighted by studies that show that removing or blocking the CD200-CD200R1 axis results in elevated myeloid activation [155]. Considering the expression of CD200 on neurons and CD200R1 on microglia, multiple studies have investigated the CD200-CD200R1 axis in the context of neuroinflammatory diseases like PD and have identified that CD200R1 inhibition augmented dopaminergic loss and neuroinflammation in the MPTP and 6-hydroxydopamine (6-OHDA) rodent models of PD [148, 156]. These studies highlight the potential of targeting endogenous negative regulators of inflammation in PD. Below we discuss a lesser-known regulator of inflammatory responses which our group and others have been investigating for its role in central and peripheral immune and inflammatory responses that affect brain health.

### RGS10

**Fig. 4:**
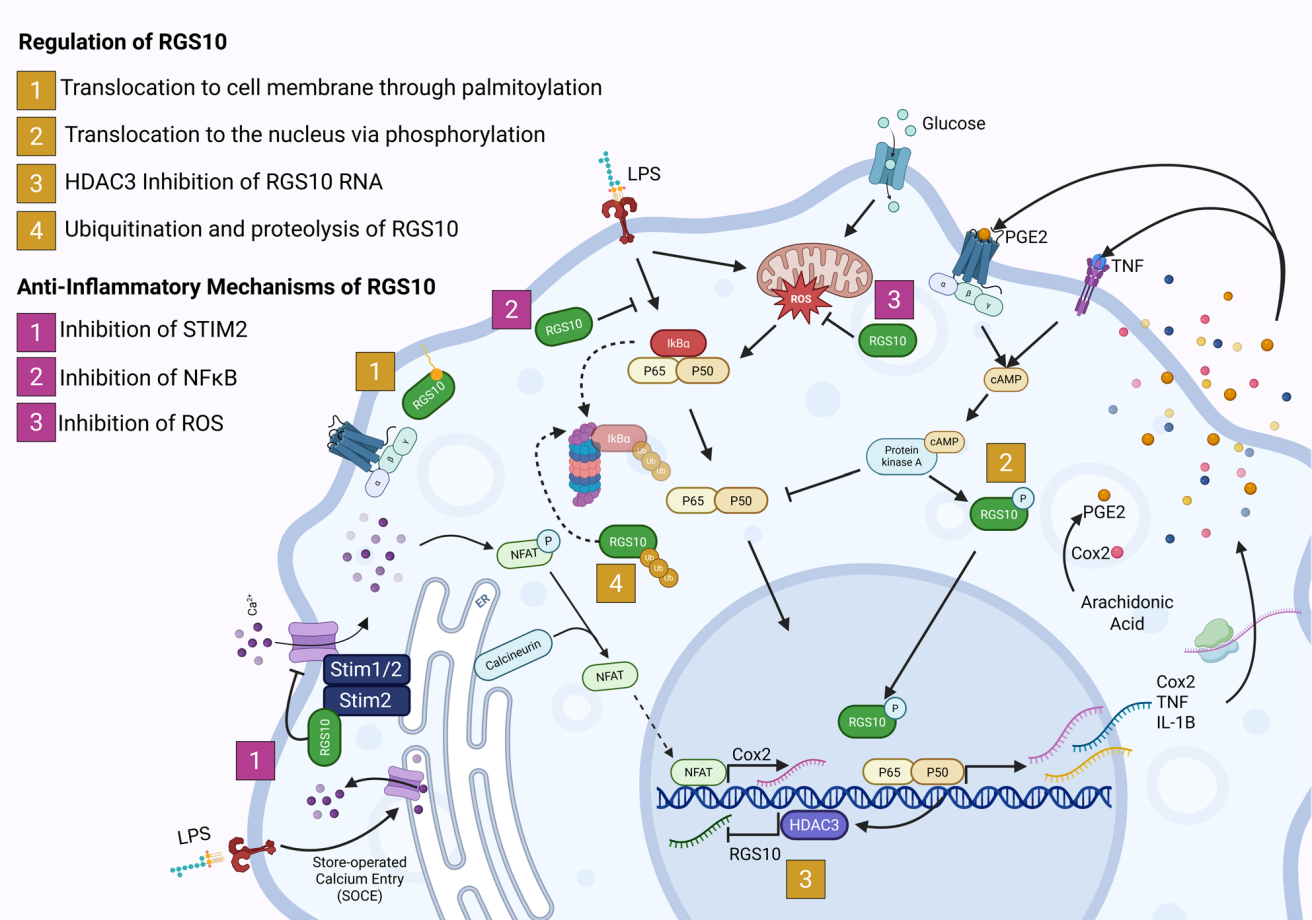
Regulation and signaling of RGS10. Yellow numbered boxes highlight intracellular regulation of RGS10. (1) Translocation of RGS10 to the cell membrane through palmitoylation. (2) Translocation of RGS10 to the nucleus via cytokine induced CAMP-PKA phosphorylation. (3) Transcriptional silencing of RGS10 due to LPS mediate NFκB induced HDAC activity. (4) Ubiquitination of RGS10 for proteasomal degradation. Pink numbered boxes highlight Anti-Inflammatory mechanism of RGS10. (1) RGS10 inhibits Stim2 mediated store operated calcium entry (SOCE) induction of Cox2. (2) RGS10 inhibits the NFκB pathway. The exact intracellular mechanism has yet to be elucidated. (3) RGS10 inhibits LPS and glucose induced production of ROS. Created with BioRender.com

Evidence suggests that regulator of G protein signaling 10 (RGS10) may act as a protective factor in the immune system. RGS10 was originally discovered as an inhibitor of G-protein coupled signaling due to its canonical GTPase activity for GPCR G_i/o_ alpha subunits [157, 158]. Interestingly, despite its canonical role as an RGS protein, RGS10 is found throughout the cell, not exclusively at the plasma membrane, and is most abundant in the cytosol and nucleus [18, 159]. RGS10 has a host of post translation modifications that impact its localization throughout the cell (Fig. 4) [160, 161]. Specifically, RGS10 targets the plasma membrane by palmitoylation,​ while phosphorylation of RGS10 on serine168 at its C terminus by cAMP-dependent PKA triggers translocation of RGS10 from the cytosol to the nucleus in HEK293 cells [160].​ The translocation of RGS10 from the cytosol to the nucleus can be observed as a result of inflammatory stimulus in primary microglia as well as forskolin stimulation of cAMP in Cos-7 fibroblast cells which suggests that nuclear translocation of RGS10 is likely a prominent regulatory mechanism to inhibit cytosolic and GAP functions of RGS10 [20, 162]. Very little work has been done to assess the nuclear functions of RGS10. However, work from the Hooks lab indicates that RGS10 interacts with multiple DNA binding proteins and may sit on promoter regions for important inflammatory cytokines such as TNF and IL-1β but quickly dissociates from these regions of chromatin upon inflammatory stimuli [18, 163]. Moreover, RGS10 is epigenetically regulated by histone deacetylation as well as DNA methylation [164–166]. Importantly, inflammatory insults such as LPS induce downregulation of RGS10 via histone deacetylation [165]. Additionally, RGS10 is a substrate for ubiquitination by the E3 ubiquitin ligase tripartite motif protein 32 (TRIM32) [167]. As indicated by its presence away from the plasma membrane, RGS10 has also been implicated in a host of non-canonical pathways and protein-protein interactions, the most well studied of which underline the role of RGS10 in immune function [18, 19].

Accordingly, RGS10 is most highly expressed in lymphoid tissues and immune cells [16, 150, 168]. Specifically, tissue resident macrophages, monocytes, and T cells express the highest levels of RGS10 [162, 169, 170]. RGS10 expression is comparatively lower in dendritic cells, B cells, and natural killer (NK) cells and has almost no expression in neutrophils [162, 170, 171]. Within T cell subsets, RGS10 is most highly expressed in naïve T cells compared to central memory or effector memory cells [162, 170]. We can appreciate a cell-type specific role for RGS10-mediated functions in immune cells, such as cytokine release and phagocytosis in myeloid cells, chemotaxis and adhesion in T and B cells, as well as activation of platelet cells [16, 21, 172, 173]. Multiple lines of evidence have identified RGS10 as a negative regulator of inflammatory responses through several pathways such as suppression of NFκB activity, STIM2 mediated calcium entry, and glycolytic production of ROS in myeloid cells [17, 18, 174–176]. As such, studies have repeatedly shown that loss of RGS10 results in hyperinflammatory conditions and dysregulated recruitment of immune cells which can increase tissue damage [17, 19, 21, 175–177]. Consistent with this, studies have revealed that peripheral RGS10 is especially important in limiting the severity of diseases where inflammation centrally dictates development and progression of pathology [16, 21, 176–180]. Specifically, RGS10 has been implicated in multiple diseases associated with aging and chronic inflammation such as obesity, heart failure, cancer, osteopetrosis, periodontitis, rheumatoid arthritis, colitis, and PD [150].

### RGS10 in peripheral disease pathogenesis

Within the past 10 years, studies have increasingly investigated the role of RGS10 in CIDs and inflammatory disease models (Table 1). From these reports, we find that RGS10 deficiency results in exaggerated metabolic dysfunction and weight gain in mice fed a high-fat diet, escalated disease activity index scores in a model of DSS induced colitis, amplified bone destruction and inflammation in periodontitis and rheumatoid arthritis mouse models, as well as increased viral titers, mortality, leukocyte infiltration, and cytokine secretion in influenza infection [175–178–179]. Outside of disease models, RGS10-deficient mice also display increased intestinal inflammation highlighting a hyperinflammatory baseline response [178]. Recently, a study also identified that RGS10 protein and mRNA were elevated in the inflamed mucosa and CD4 + T cells of individuals with ulcerative colitis compared to healthy controls, interestingly this study also reported ameliorated disease activity index scores for DSS induced colitis with RGS10 KO [181]. Here as with other autoimmune conditions, evidence supports that RGS10 promotes the differentiation of and trafficking of Th1/Th17 T cells which could account for ameliorated phenotypes with RGS10 deficiency [173, 181]. These studies highlight the cell specific functions of RGS10 that may impact inflammatory vs. autoimmune conditions differently. Moreover, the cancer field has illuminated that inflammation can not only predispose tissues for the development of cancer but also promote tumorigenesis at every stage [182]. Interestingly, changes in RGS10 expression due to DNA methyltransferase epigenetic suppression have also been correlated to poor prognosis in multiple cancers including colorectal carcinoma, hepatocellular carcinoma, ovarian cancer, and pediatric acute myeloid leukemia [150, 164, 183]. RGS10 has also been implicated in distant metastasis of breast cancer [184]. Furthermore, case studies have reported that rare genetic mutations in RGS10 in humans could be associated with immunodeficiency and persistent viral infection [162]. Patients with RGS10 mutations were also found to have increased IgG and IgM antibodies with normal response to immunization antigens but decreased response to polysaccharide antigens, defective lymphocyte chemotaxis, and lymphoid tissue abnormalities [162].

**Table 1:** RGS10 in peripheral inflammatory diseases

Condition	Study	Organism/Model	RGS10 Alteration or manipulation	Conclusions
Metabolic Dysfunction	Fang, Chung [179]	C57B6 Mice - High fat Diet Induced metabolic Dysfunction	RGS10 KO mice	↑ RGS10 KO Weight gain, Insulin resistance, and inflammation compared to WT
Colitis	Houser, Caudle [178]	C57B6 Mice – DSS induced Colitis	RGS10 KO mice	↑ RGS10 KO Disease activity Index scores compared to WT
Colitis	Yang, Shao [181]	Human – Ulcerative colitis	Lentiviral suppression and overexpression of RGS10	↑ RGS10 in UC colonic biopsies compared to control ↑RGS10 mRNA in CD4 + T Cells exposed to proinflammatory cytokines ↑ Th1 and TH17 T cell differentiation and pro-inflammatory profiles with RGS10 overexpression in CD4 + T cells
		C57B6 Mice – DSS induced Colitis	RGS10 KO mice	↓ RGS10 KO Disease activity Index scores compared to WT ↓ Th1/Th17 cells in DSS-induced colitis RGS10 KO mice compared to WT
Rheumatoid Arthritis	Ren, Wei [176]	DBA/J1 Mice – Collagen Induced Arthritis	AAV-Sh-RGS10 downregulation	↑ RA severity and inflammation with downregulation of RGS10 compared to control
Rheumatoid Arthritis and Periodontitis	Chan, Tan [174]	DBA/J1 Mice – Collagen Induced Arthritis and P. Gingivalis Induced Periodontitis	AAV-Sh-RGS10 downregulation	↑ Periodontitis and pro-inflammatory signaling with RGS10 downregulation in treatment groups compared to control
Periodontitis	Wei, Li [175]	BALB/c Mice – Periapical Periodontitis	AAV-Sh-RGS10 downregulation	↓ Alveolar bone destruction with RGS10 downregulation ↑ NFκB pro-inflammatory signaling with RGS10 downregulation
Periodontitis	Li, Yue [185]	BALB/c Mice – Apical Periodontitis	AAV-RGS10 overexpression	↓ Alveolar bone destruction and macrophage infiltration with local RGS10 overexpression
Viral Infection	Almutairi, Sarr [177]	C57B6 mice – Lethal Influenza A infection	RGS10 KO mouse	↑ RGS10 KO Weight loss, mortality, lung inflammation, myeloid leukocyte infiltration into the lungs, and neutrophil activation compared to WT
Systemic Inflammation	Jernigan, Staley [186]	C57B6 Mice - CSI model:1 × 10^6^ Eu/mL LPS twice a week for 6 weeks	RGS10 KO Mice	↑ Inflammatory myeloid cells, cytotoxic T cells in the blood of LPS treated RGS10 KO mice compared to WT ↓MHCII+ antigen presenting cell types in the blood of LPS RGS10 KO mice compared to WT
Cancer (Ovarian)	Hu, Zheng [187]	Human - Databases	N/A	↑RGS10 expression in Ovarian Cancer ↑RGS10 expression positively correlated with the Immune Score of multiple cancers
Cancer (Ovarian)	Kucuk, Kibar [188]	Human – Databases Cell Line – Ovarian cancer	N/A	↑RGS10 expression in Ovarian Cancer ↓RGS10 expression with chemotherapy
Cancer (Ovarian)	Ali, Cacan [164] Hooks, Callihan [189]	Human – Databases Cell Line – Ovarian cancer	N/A	↓RGS10 expression with acquired chemoresistance
Cancer (Breast)	Liu, Jiang [184]	Humans – Breast Cancer	N/A	↓RGS10 expression in Breast Cancer ↓RGS10 predicted worse prognosis in patients with breast cancer
Cancer (Colorectal)	Caldiran and Cacan [183]	Human - Dataset	N/A	↓RGS10 due to DNA methylation was associated with low survival rates in colorectal carcinoma
Cancer (Hepatocellular carcinoma)	Wen, Li [190]	Human -Hepatocellular carcinoma	N/A	↑Methylation of RGS10 in Hepatocellular carcinoma
Cancer (Pediatric acute myeloid leukemia)	Chaudhury, O’Connor [191]	Human - Dataset	N/A	↑RGS10 gene expression in acute myeloid leukemia correlated with poor clinical outcomes
Immunodeficiency	Chinn, Xie [162]	Human – Family case study	Rare RGS10 mutation *Compounding PiзK Mutation	↑ Persistent Infection ↓ lymphocyte chemotaxis Abnormal lymphoid organ architecture ↓Decreased stature

### RGS10 in CNS disease pathogenesis

Outside of lymphoid tissues, RGS10 has also been shown to be expressed throughout the brain, in areas such as the hippocampus, striatum, and dorsal raphe [159, 192]. Particular types of neurons including forebrain interneurons, pyramidal cells, enkephalin expressing neurons, and newly born neurons within the dentate gyrus preferentially expressed RGS10, with RGS10 being found in pre- and postsynaptic locations as well as in the cytoplasm and nucleus [159, 192, 193]. While studies have confirmed that RGS10 continues to play its role as a GAP protein in neurons by attenuating inhibitory GPCR activity such as mu opioid receptor activity, the subcellular location of RGS10 within the brain suggests a larger role for RGS10 at the synapse and perhaps in gene expression activity [194]. Recent studies have also identified RGS10 in astrocytes, with RGS10 levels increasing with neuroprotective carbon monoxide treatment, while the protein atlas has identified high levels of RGS10 expression in Schwann cells, which are responsible for peripheral nerve myelination [168, 170, 195]. Single nucleotide polymorphisms (SNPs) in and around RGS10 have been implicated in multiple neurologic conditions including schizophrenia, depression, and suicide [196, 197]. However, analysis of human postmortem brain tissue from suicide victims, patients diagnosed with major depressive disorder antemortem, and patients diagnosed with schizophrenia antemortem revealed no differences in the subcellular location or amount of RGS10 between groups [198]. Furthermore, a SNP in GRK5/RGS10 has been weakly associated with age related maculopathy and RGS10 transcript was found to be a hub gene for protein-protein interaction network of immune related differentially expressed genes in diabetic retinopathy [199, 200]. Changes in RGS10 levels also occur in autism like behaviors, a mouse model of down syndrome, and as a result of seizure activity and dopaminergic depletion with reserpine but not 6-hydroxydopamine [167, 201–203]. RGS10 has also been shown to be protective of dopaminergic neurons in the face of TNF mediated toxicity through the PKA/cAMP pathway [204].

Importantly, RGS10 is ubiquitously expressed in the innate immune cells of the brain, microglia [159]. RGS10 has been identified as a homeostatic marker of microglia, mitigating hyper-proinflammatory responses under disease associated conditions, indicating its critical role in regulating the immune response of the CNS [19, 205, 206]. Moreover, RGS10 may also play a role in peripheral immune cell population trafficking and dynamics in the CNS. In RGS10 deficient mice, basal levels of peripheral immune cell infiltration in the brain were observed [207]. RGS10 KO mice also display altered peripheral immune cell infiltration frequencies in aging [207]. Specifically, young KO mice have decreased frequencies of monocytes and microglia but increased frequencies of granulocytes and CD8 + T cells in the brain [207]. Aged RGS10 KO mice, conversely, do not have significantly different infiltrating immune cell frequencies compared to aged WT mice [207]. Interestingly, there is evidence that RGS10 levels decrease in microglia and peripheral immune cells with age and significant inflammatory insults [186, 207–209]. Conversely, RGS10 levels rise with age in peripheral monocytes and granulocytes [207]. However, the level of RGS10 was not measured in CNS-associated peripheral immune cells which may prove to be different, as immune cell phenotypes are extremely context dependent [207]. Within a CSI mouse model, loss of RGS10, induced a bias for inflammatory myeloid cells and cytotoxic T cell subsets while reducing MHCII+ antigen presenting cells in circulation and in and around the brain [186]. As such, RGS10 is an important homeostatic regulator of the immune system in the CNS.

With the growing knowledge of inflammation in CNS related diseases it is not surprising that RGS10 has also been implicated in multiple neurodegenerative diseases including PD and MS [17, 19, 20, 22, 173]. In the experimental autoimmune encephalomyelitis (EAE) mouse model of MS, RGS10 KO was found to be protective, likely through decreased infiltration of peripheral immune cells demonstrated by decreased CD3+, CD11b+, and CD45^high^ populations in the CNS [173]. Decreased peripheral immune cell infiltration was not a result of impaired antigen presentation from dendritic cells; however, it may have been mediated through decreased chemotaxis and adhesion seen in RGS10 deficient Th1 cells [173]. Conversely, colitis and RGS10 deficiency was found to increase CD8 + mRNA expression in the substantia nigra [178]. These studies indicate that RGS10 regulates CNS peripheral immune cell infiltration in a disease and cell specific manner and may represent a potential mechanism of RGS10 mediated peripheral influence on the CNS.

Importantly, multiple studies have elucidated RGS10 as a neuroprotective agent in the development of clinically relevant PD pathologies [17, 20, 178, 204]. Several of these studies revealed a synergistic relationship between peripheral inflammatory insults and RGS10 deficiency in the development of PD pathology [20, 178]. One such study revealed that RGS10 deficient mice exposed to CSI developed dopaminergic degeneration in the substantia nigra as well microgliosis [20]. Moreover, introducing RGS10 into the midbrains of hemi-parkinsonian rats prevents the loss of dopaminergic neurons in the substantia nigra and the induction of microgliosis in a 6-OHDA model of nigrostriatal degeneration [17]. Lastly, RGS10 deficiency synergistically exacerbated colitis and sub-threshold MPTP induced parkinsonian neuropathology [178]. These studies indicate a neuroprotective role for RGS10 in chronic inflammation and nigrostriatal neurodegeneration likely through its role in suppression of inflammation and orchestration of a balanced immune response (Table 2). Moreover, we find that RGS10-deficient mice display intestinal inflammation and increased expression of synuclein in the gut similar to that in PD patients [178]. Furthermore, RGS10 is decreased in both the CSF as well as subsets of circulating peripheral immune cells in PD patients, implicating RGS10 as a clinically relevant immunological target [178, 186].

**Table 2:** RGS10 in neurodegenerative diseases

Disease	Study Reference	Organism/Model	RGS10 Alteration or manipulation	Outcome
Parkinson’s Disease	Lee, McCoy [20]	C57B6 Mice - CSI model:7.5 × 10^5^ Eu/mL LPS twice a week for 6 weeks	RGS10 KO mice (mixed background)	↑Microgliosis with RGS10 KO compared to control ↓Tyrosine Hydroxylase in the nigra of RGS10 Ko compared to control
Parkinson’s Disease	Lee, Chung [17]	C57B6 Mouse	RGS10 KO mice	↑ NFκB activity, Proinflammatory cytokine secretion, and midbrain dopaminergic cell line cytotoxicity from RGS10 KO primary microglia
			Lenti Viral RGS10 Overexpression	↓ Proinflammatory cytokine secretion and midbrain dopaminergic cell line cytotoxicity with RGS10 overexpression compared to controls in primary microglia
		Rat – PD model: 6 OHDA PD model	Lenti Viral RGS10 Overexpression	↓Microgliosis with RGS10 overexpression compared to control ↑Tyrosine Hydroxylase in the nigra of RGS10 overexpression compared to control
Parkinson’s Disease	Houser, Caudle [178]	Human – Parkinson’s Disease	N/A	↓ RGS10 in PD CD16 + monocytes, Cd16 + Cd14+ Monocytes, and CD4 + T cells compared to controls
		C57B6 Mice - DSS induced colitis and PD Model: sub-threshold MPTP	RGS10 KO Mice	↓ Tyrosine Hydroxylase in the nigra of RGS10 KO DSS + MPTP mice compared to DSS or MPTP alone
Parkinson’s Disease	Jernigan, Staley [186]	Human- Parkinson’s Disease	N/A	↓RGS10 in CSF of individuals with Parkinson’s Disease
		C57B6 Mice - CSI model:1 × 10^6^ Eu/mL LPS twice a week for 6 weeks	RGS10 KO Mice	↑ Inflammatory myeloid cells, cytotoxic T cells in and around the brain of LPS treated RGS10 KO mice compared to WT ↓MHCII+ antigen presenting cell types in and around the brain of LPS RGS10 KO mice compare to WT ↑Dendritic cells in and around the brains of RGS10 KO mice compared to WT
Multiple Sclerosis	Lee, Kannarkat [173]	C57B6 Mice -EAE	RGS10 KO mice	↓Disease incidence, severity, and onset compared to controls ↓Leukocyte chemotaxis to the CNS compared to controls ↓Disease attenuation with adoptive transfer of RGS10 KO TH1 T cells compared to controls

## Conclusions and future directions

Here we have described the development and classification of CSI, particularly CSI in NDs such as PD. We highlight that risk factors for developing NDs are all common sources of CSI, and that CSI is present in multiple NDs. Additionally, we have reviewed the current state of pre-clinical models for CSI and their respective limitations in neurodegenerative studies. Moreover, we detail the cellular mechanisms behind CSI including multiple routes of immune dysregulation and how CSI can impact the CNS. Overall, further investigation is required to know the extent to which CSI contributes to the development and progression of NDs in patients, however the work reviewed here highlights CSI as a highly relevant and central target for clinical intervention. Lastly, we feature RGS10 as an important mediator of CSI and the development of NDs.

Critically, the field needs to develop a standardized pre-clinical model of CSI. Ideally, a standardized paradigm would be developed around the use of precise endotoxin units, comparable levels of low-grade inflammation in aged mice to that seen in aged humans, as well as meaningful time spans for the chronicity of the inflammatory insult. In conjunction, longitudinal studies in humans are necessary to determine comparable levels of inflammation and whether it is present prior to the onset of ND pathology and diagnosis. Moreover, it is prudent to parse apart the extent to which the accumulation of particular risk factors of NDs/sources of CSI, advance CSI alone and in conjunction with one another, as well as their respective impact on ND pathology. Crucially, these studies should be followed by experiments in which the development of CSI is inhibited to understand if it is required for the development of ND pathology and if it is, which aspect of CSI contributes the most. Moreover, future work regarding RGS10 should aim to broaden our understanding of which neurodegenerative diseases RGS10 may be altered in and how it may impact disease pathogenesis in a cell specific manner. Lastly, additional studies should be dedicated to understanding the interactome of RGS10 in a cell specific manner to better elucidate intracellular regulators and mechanisms of RGS10 in immune cells. In conclusion, future work in CSI has the potential to identify novel and effective preventative strategies to treat CSI and mitigate the risk for age-related NDs. Accordingly, targeting immunoregulatory proteins, such as RGS10, may prove to be an efficient risk mitigation strategy for the development and progression of PD and other CIDs. 

## Data Availability

No datasets were generated or analysed during the current study.

## Abbreviations

CSI: Chronic systemic inflammation
ND: Neurodegenerative disease
RGS10: Regulator of G protein signaling 10
AD: Alzheimer’s disease
PD: Parkinson’s disease
PAMPs: Pathogen associated molecular patterns
DAMPs: Disease associated molecular patterns
ROS: Reactive oxygen species
CXCL9: Chemokine ligand 9
CRP: C reactive protein
TNF: Tumor necrosis factor
CSF: Cerebral spinal fluid
CNS: Central nervous system
CIDs: Chronic inflammatory diseases
RA: Rheumatoid arthritis
IBD: Inflammatory bowel disease
IBS: Irritable bowel syndrome
LPS: Lipopolysaccharide
IV: Intravenous
IP: Intraperitoneal
WT: Wild type
NET: Neutrophil extracellular traps
APC: Antigen presenting cell
IL4: Interleukin 4
IL-1RA: Interleukin 1 receptor antagonist
IL-10: Interleukin 10
PUFA: Polyunsaturated fatty acids
SPMs: Special pro-resolving mediators
EPA: Eicosapentaenoic acid
DPA: Docosapentaenoic acid
DHA: Docosahexaenoic acid
COX-2: Cyclooxygenase-2
LOX: Lipoxygenase
NSAID: Non-steroidal anti-inflammatory drug
IL-13: Interleukin 13
CHIP: Clonal hematopoiesis of indeterminate potential
PD1: Programmed Cell Death 1
TIM3: T cell immunoglobulin and mucin-domain containing 3
PET: Positron emission tomography
DCE-MRI: Dynamic contrast enhanced magnetic resonance imaging
IL-6: Interleukin 6
IL-8: Interleukin 8
HSCs: Hematopoietic stem cells
SASP: Senescence associated secretory phenotype
HDACS: Histone Deacetylases
HATS: Histone Acetyltransferases
NFκB: Nuclear factor kappa-light-chain-enhancer of activated B cells
BBB: Blood brain barrier
GMCSF: Human granulocyte macrophage colony stimulating factor
ERK: Extracellular signal-regulated kinase
CCR3: C-C chemokine receptor type 3
NLRP3: NLR family pyrin domain containing 3
UPDRS: Unified Parkinson’s disease rating scale
TNFAIP3: Tumor necrosis factor α-induced protein 3
IL-1β: Interleukin 1 beta
MPTP: 1-Methyl-4-phenyl-1,2,3,6-tetrahydropyridine
6-OHDA: 6-hydroxydopamine
mTOR: Mechanistic Target of Rapamycin
GPCR: G protein coupled receptor
cAMP: Cyclic AMP
PKA: Protein kinase A
GAP: GTPase-activating protein
TRIM32: Tripartite motif protein 32
NK: Natural killer
STIM2: Stromal interaction molecule 2
DSS: Dextran sodium sulfate
SNP: Single nucleotide polymorphism
KO: Knock out
MS: Multiple Sclerosis
EAE: Experimental autoimmune encephalomyelitis
